# Are bone erosions detected by magnetic resonance imaging and ultrasonography true erosions? A comparison with computed tomography in rheumatoid arthritis metacarpophalangeal joints

**DOI:** 10.1186/ar1995

**Published:** 2006-07-18

**Authors:** Uffe Møller Døhn, Bo J Ejbjerg, Michel Court-Payen, Maria Hasselquist, Eva Narvestad, Marcin Szkudlarek, Jakob M Møller, Henrik S Thomsen, Mikkel Østergaard

**Affiliations:** 1 Department of Rheumatology, University of Copenhagen Hvidovre Hospital, Hvidovre, Denmark; 2 Department of Radiology, University of Copenhagen Rigshospitalet, Copenhagen, Denmark; 3 Department of Diagnostic Radiology, University of Copenhagen Herlev Hospital, Herlev, Denmark; 4 Department of Rheumatology, University of Copenhagen Herlev Hospital, Herlev, Denmark

## Abstract

The objective of the study was, with multidetector computed tomography (CT) as the reference method, to determine whether bone erosions in rheumatoid arthritis (RA) metacarpophalangeal (MCP) joints detected with magnetic resonance imaging (MRI) and ultrasonography (US), but not with radiography, represent true erosive changes. We included 17 RA patients with at least one, previously detected, radiographically invisible MCP joint MRI erosion, and four healthy control individuals. They all underwent CT, MRI, US and radiography of the 2nd to 5th MCP joints of one hand on the same day. Each imaging modality was evaluated for the presence of bone erosions in each MCP joint quadrant. In total, 336 quadrants were examined. The sensitivity, specificity and accuracy, respectively, for detecting bone erosions (with CT as the reference method) were 19%, 100% and 81% for radiography; 68%, 96% and 89% for MRI; and 42%, 91% and 80% for US. When the 16 quadrants with radiographic erosions were excluded from the analysis, similar values for MRI (65%, 96% and 90%) and US (30%, 92% and 80%) were obtained. CT and MRI detected at least one erosion in all patients but none in control individuals. US detected at least one erosion in 15 patients, however, erosion-like changes were seen on US in all control individuals. Nine patients had no erosions on radiography. In conclusion, with CT as the reference method, MRI and US exhibited high specificities (96% and 91%, respectively) in detecting bone erosions in RA MCP joints, even in the radiographically non-erosive joints (96% and 92%). The moderate sensitivities indicate that even more erosions than are seen on MRI and, particularly, US are present. Radiography exhibited high specificity (100%) but low sensitivity (19%). The present study strongly indicates that bone erosions, detected with MRI and US in RA patients, represent a loss of calcified tissue with cortical destruction, and therefore can be considered true bone erosions.

## Introduction

Radiography is the mainstay of the evaluation of structural joint damage in patients with rheumatoid arthritis (RA). It is a routine procedure for diagnosis and prognostication in RA patients, and is an important end-point in clinical trials [1,2]. Detection of bone erosions at the time of RA diagnosis is related to a poor long-term functional and radiographic outcome [3-7], and the presence of erosions in early undifferentiated arthritis is a risk factor for developing persistent arthritis [8]. For these reasons, earlier detection of bone erosions, using any imaging modality, would be expected to be of considerable clinical importance. Unfortunately, radiography does not permit visualization of the earliest stages of erosive changes in RA, and other imaging modalities have emerged as methods permitting improved visualization of early bone erosions [9-12].

Magnetic resonance imaging (MRI) has been demonstrated to be more sensitive than radiography in detecting erosive changes in RA, especially the subtle changes that occur in early disease [9-11,13,14]. Furthermore, MRI has the ability to visualize synovitis, which is the primary pathologic process in RA joint involvement [14-16], and bone oedema, which is a strong predictor of future erosive bone changes [17-19].

Ultrasonography (US), although less validated, has been reported to be more sensitive than radiography and comparable to MRI in detecting bone erosions in RA metacarpophalangeal (MCP) [10,20] and metatarsophalangeal joints [21]. US has great site dependency, exhibiting the highest sensitivity in detecting bone erosions at the easily accessible joints such as the 2nd and 5th MCP joints and the proximal interphalangeal joints [20,22]. Additionally, with US it is possible to visualize soft tissue changes and synovitis, using gray-scale US and Doppler US techniques [23-25].

Conventional radiography is based on attenuation of X-rays, and calcified tissues such as bone are readily depicted because of their markedly greater attenuation in comparison with the surrounding soft tissues. Because imaging with MRI and US does not depend on X-rays, it has been speculated to which extent erosions detected using these modalities reflects true loss of calcified tissue, that is, are true erosions[26,27].

Computed tomography (CT) is a tomographic radiographic imaging method that visualizes calcified tissue with high resolution, and CT can be considered a standard reference for detecting destructions of calcified tissue, such as bone erosions in RA [12,26]. By using multidetector CT with multiplanar reconstruction, three-dimensional visualization of joints is possible, whereas radiography is a projection technique offering only a two-dimensional visualization of the three-dimensional anatomy. However, in comparison with MRI and US, CT inadequately visualizes soft tissue changes.

No comparative studies of CT, MRI, US and radiography in evaluating erosive bone changes in RA MCP joints have been reported. Although one study compared CT and MRI with respect to their ability to evaluate bone erosions in RA wrists [12], data from comparative studies with CT are sparse, and it remains unclear whether erosions seen on MRI and US, but not on radiography, represent true destructive bone changes. Therefore the main objective of the present study was to investigate whether bone erosions detected using MRI and US represent bone loss, including cortical destruction, and therefore are true erosive changes. In this cross-sectional methodological study, we used CT as the standard reference method in order to determine the sensitivity, specificity and accuracy of MRI, US and radiography in detecting bone erosions in RA MCP joints.

## Materials and methods

### Patients and control individuals

Seventeen patients with RA fulfilling American College of Rheumatology 1987 criteria [28] and four healthy control individuals underwent CT, MRI, US and radiography of the 2nd to 5th MCP joints of one hand on the same day (details on patients and control individuals are given in Table 1).

Patients were recruited from the Department of Rheumatology, Copenhagen University Hospital at Hvidovre. All patients were selected from former MRI studies, and were eligible to participate in the study if they, in at least one of the examined MCP joints, at a previous examination had at least one radiographically invisible MRI lesion, presumed to be an erosion. All imaging procedures were performed at the Department of Radiology, Copenhagen University Hospital at Herlev.

The study was approved by the local ethics committee, and written informed consent was obtained from all participants.

### Computed tomography

**Figure 1:**
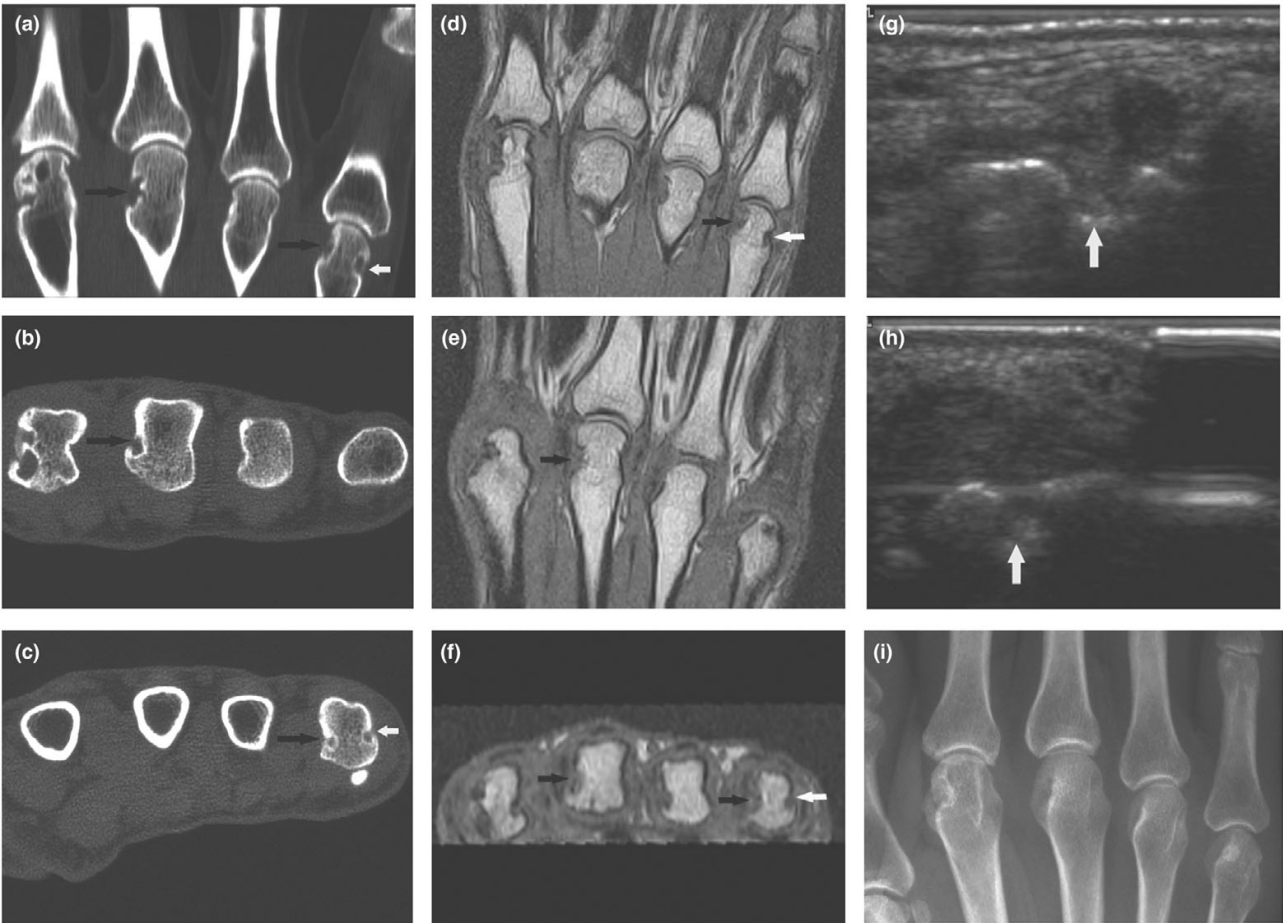
CT, MRI, US and radiography of a RA patient's 2nd to 5th MCP joints. CT of the 2nd to 5th MCP joints, in **(a) **coronal and **(b, c) **axial planes. Erosions in the 3rd and 5th metacarpal heads are marked with arrows. T1-weighted magnetic resonance images of the 2nd to 5th MCP joints, in the **(d, e) **coronal and **(f) **axial planes reveal the same erosions in the 3rd and 5th metacarpal heads as marked on the CT images. US at the ulnar aspect of the 5th metacarpal head, in **(g) **longitudinal and **(h) **transversal planes. An erosion (white arrow) at the same site as detected by CT and MRI (white arrows in panels a, c, d and f) is documented in both planes. **(i) **Radiography reveals no erosions at the corresponding sites. CT, computed tomography; MCP, metacarpophalangeal; MRI, magneticresonance imaging; RA, rheumatoid arthritis; US, ultrasonography.

**Figure 2:**
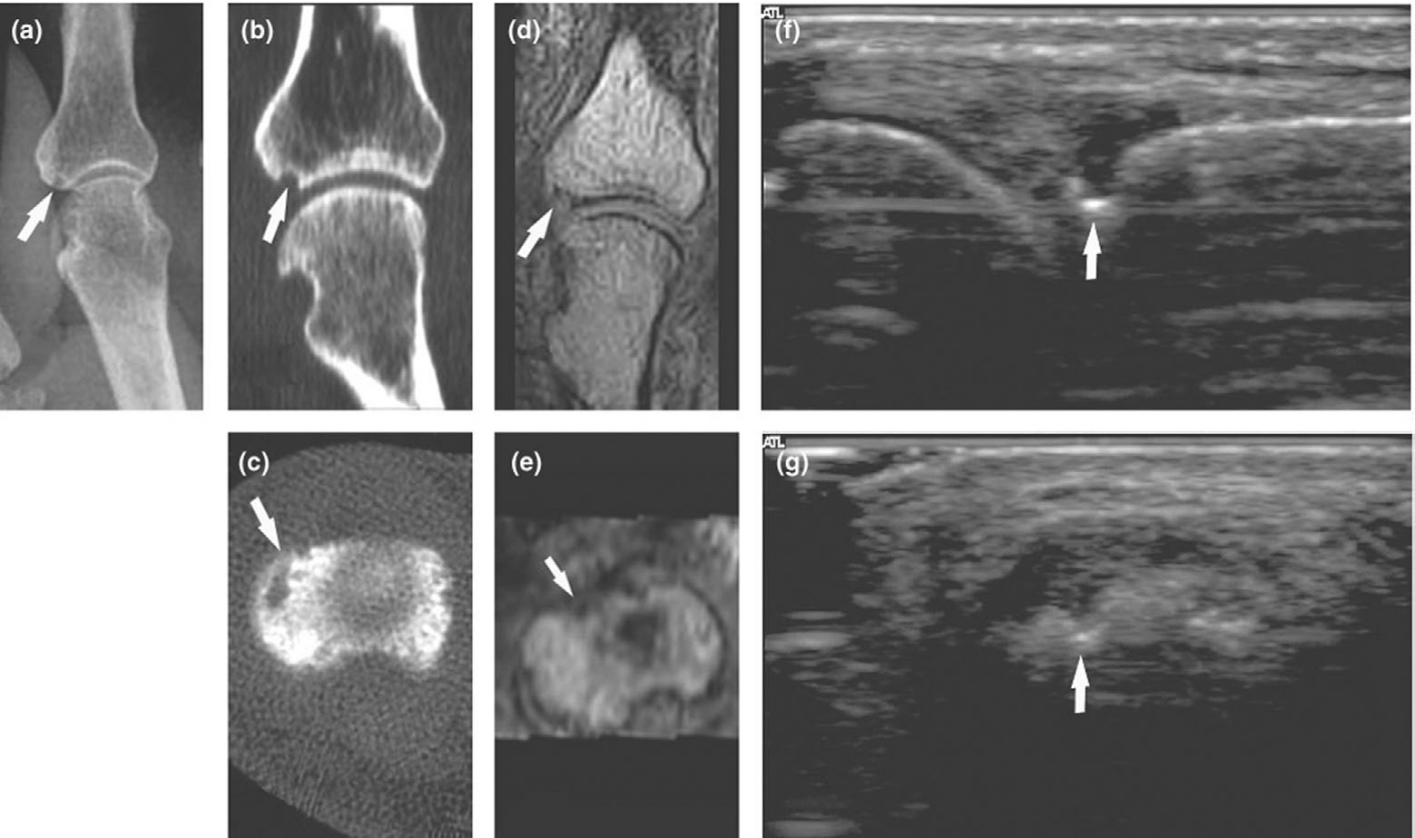
Radiography, CT, MRI and US of a RA patient's 2nd MCP joint. **(a) **Radiography in anteroposterior projection. CT in **(b) **coronal and **(c) **axial planes. T1-weighted MRIin **(d) **coronal and **(e) **axial planes. US in **(f) **longitudinal and **(g) **transversal planes. Anerosion (white arrows) at the base of the 2nd proximal phalanx isvisualized on radiography (panel a), CT (panels b and c) andultrasonography (panels f and g) in both planes. This erosion was notscored on MRI. If the corresponding area on MRI (panels d and e) isreassessed, then the reader gets the impression of the presence of anerosion, with the same configuration as on CT and radiography. CT, computed tomography; MCP, metacarpophalangeal; MRI, magnetic resonance imaging; RA, rheumatoid arthritis; US, ultrasonography.

A Philips Mx8000 IDT multidetector unit (Philips Medical Systems; Cleveland, Ohio, USA) was used for all examinations (parameters: 90 kV, 100 mAs, pitch 0.4 mm, slice spacing 0.4 mm, overlap 50%). Patients were placed in the prone position with the arm stretched and the palm facing down. Images with a voxel size of 0.4 × 0.4 × 1.0 mm were obtained, and software for multiplanar reconstruction created axial and coronal reconstructions with a slice thickness of 1.0 mm (slice spacing 0 mm, overlap 0 mm), and these were used for image evaluation (Figures 1 and 2). In order to assess the interobserver agreement, CT images were evaluated independently by two of the investigators: a musculoskeletal radiologist (MH) and a rheumatologist (MØ) with experience from previous studies in evaluating magnetic resonance images of RA finger joints. Prior to the evaluation, it was decided that the scoring by MØ would be used for comparison with results of the other imaging modalities.

### Magnetic resonance imaging

A Philips Panorama 0.6 T unit (Philips Medical Systems; Helsinki, Finland), using a receive-only three channel phased solenoid coil, was used for all examinations. Patients were placed in the supine position, with the hand alongside the body and the palm facing the body. Acquired images included a coronal T1-weighted three-dimensional fast field echo (repetition time 20 ms, echo time 8 ms, flip angle 25°, voxel size 0.4 × 0.4 × 0.4 mm, matrix 216 × 216 pixels, number of acquisitions 2, acquisition time 5.23 min). Multiplanar reconstructions of the T1-weighted three-dimensional fast field echo sequence were done in the axial and coronal planes with a slice thickness of 0.4 mm, and these were used for image evaluation (Figures 1 and 2). Magnetic resonance images were evaluated by a rheumatologist (BJE) with experience from previous studies in evaluating magnetic resonance images of RA finger joints.

### Ultrasonography

US was performed by a musculoskeletal radiologist (MC-P) with experience in US of RA joints from previous studies. The same Philips 5000 HDI unit (Philips Medical Systems; Bothell, Washington, USA) with a 15-7 MHz linear array hockey stick transducer was used for all examinations. The dorsal and palmar aspects of the 2nd to 5th MCP joints, the radial aspect of the 2nd MCP joint and the ulnar aspect of the 5th MCP joint were examined with longitudinal and transversal scans (Figures 1 and 2).

### Radiography

Radiography was done on a Philips Digital Diagnost unit (Philips Medical Systems; Hamburg, Germany) with a resolution of 0.3 mm. Posterior anterior and Nørgaard [29] projections were obtained (Figures 1 and 2). The images were evaluated by a musculoskeletal radiologist (EN) with experience from previous studies in evaluating RA radiographs.

### Imaging evaluation

All imaging modalities were evaluated with investigators blinded to clinical and other imaging data. Each MCP joint quadrant (radial and ulnar part of the metacarpal head and phalangeal base, respectively) was scored for the presence or absence of erosions. The localizations of erosions were marked on a preformed scoring sheet, which allowed exact positioning of erosions in all three planes. Erosions on CT were defined as a sharply demarcated area of focal bone loss seen in two planes, with a cortical break (loss of cortex) seen in at least one plane. Definitions of MRI erosions were as suggested by Outcome Measures in Rheumatology (OMERACT) Rheumatoid Arthritis MRI Scoring System (RAMRIS) [30] (that is, a sharply marginated bone lesion, with correct juxta-articular localization and typical signal characteristics, which is visible in two planes with a cortical break seen in at least one plane). US erosions were defined as irregularities of the bone surface of the area adjacent to the joint and seen in two planes, as suggested by Szkudlarek and coworkers [31].

### Statistical analysis

With CT as the standard reference method, the sensitivity, specificity and accuracy of MRI, US and radiography were calculated. The interobserver agreement between the two readers of CT images was calculated.

## Results

In total, 336 quadrants were assessed for erosions, of which 78, 64, 55 and 16 quadrants had erosions on CT, MRI, US and radiography, respectively. Of the quadrants with erosions on MRI, US and radiography, 53, 33 and 15, respectively, could be confirmed by CT. For radiography, the overall sensitivity, specificity and accuracy were 19%, 100% and 81%, respectively. For MRI, the corresponding values were 68%, 96% and 89%, and for US they were 42%, 91% and 80% (Table 2).

In order to evaluate the performance of MRI and US in the radiographically non-erosive areas, the analysis was repeated after excluding all quadrants with radiographic erosions (16 quadrants). In this analysis MRI exhibited a sensitivity, specificity and accuracy of 65%, 96% and 90%, respectively. For ultrasonography the corresponding figures were 30%, 92% and 80% (Table 3).

To evaluate the performance of US in regions in which there was good access for visualization of bone surfaces, we repeated the analysis including just the palmar and dorsal aspects of all joints and the radial and ulnar aspect of the 2nd and 5th MCP joints, respectively. At these locations, US exhibited overall sensitivity, specificity and accuracy of 60%, 92% and 87%, respectively.

With radiography, eight out of 17 patients were judged to have at least one erosion in the examined joints, whereas all patients on CT and MRI and 15 patients on US had at least one erosion. None of the healthy control individuals had any erosions as judged by CT, MRI, or radiography, but erosion-like changes were seen on US in all healthy control individuals (eight quadrants in three MCP joints in one person, and in one quadrant each in the remaining three persons).

The concordance between readings by the two CT readers (that is, the overall agreement) was 90%.

## Discussion

The main purpose of the present study was to investigate whether erosions detected by MRI and US could be confirmed by CT (that is, whether they are true erosions). With CT as the standard reference method, high specificity of MRI and US in detecting bone erosions in RA MCP joints was demonstrated, even when only radiographically normal MCP joints (that is, the joints with the most subtle changes) were considered. This study strongly indicates that radiographically invisible bone erosions detected by MRI and US are true erosive changes.

All patients were selected from former MRI studies and were eligible to participate in the study if they had at least one radiographically invisible MRI lesion, presumed to be an erosion. This selection was made in order to include only patients whose joints were not too severely damaged, that is, the patients in which MRI and US would be expected to have the greatest clinical value. Clearly, the sensitivity of radiography, MRI and US would have been higher if joints with extensive erosive changes had been included.

Previous studies [9-11,14] have reported that radiography has poor sensitivity in detecting bone erosions compared with MRI. In the present study we also found that radiography had very poor sensitivity (19%) in detecting bone erosions in RA MCP joints compared with CT. This finding verifies that radiography, possibly because of its two-dimensional visualization of the joint, is insensitive in detecting the earliest stages of erosive bone changes in RA. In this material, radiography was unable to detect any erosions in nine out of 17 patients, whereas at least one erosion was seen on CT and MRI in all patients. When retrospectively reassessing radiographs of areas with erosions on CT, MRI and US, subtle changes (for example, changes in the trabecular pattern) may occasionally be recognized (for example, the 5th metacarpal head on Figure 1). However, such changes are still not considered erosions by current criteria for radiographic erosions.

Signal on radiography and CT is based on attenuation of X-rays, and bone and other calcified tissues are easily depicted because of markedly higher X-ray attenuation by these tissues than by the surrounding soft tissues. The signal on magnetic resonance images is not dependent on X-rays but on presence of mobile protons in the tissue, and as the water content in bone is very low cortical bone is depicted as signal voids silhouetted against signal-emitting bone marrow and periosseous tissues. It has been argued that MRI is not well suited to visualizing lesions of calcified tissue, and the nature of bone erosions visualized with MRI, that are not visible on radiography, has been questioned [26]. In this study MRI findings were in very good agreement with findings from the applied high-resolution three-dimensional tomographic X-ray modality (that is, CT findings), even in regions without radiographic erosions.

To our knowledge, no published studies have compared CT, MRI, US and radiography in small RA joints. In a recent study conducted by Perry and coworkers [12], a comparison between CT and MRI in wrist joints of nine RA patients revealed an overall agreement between CT and MRI of 87% in detecting bone erosions. As in the present study, Perry and coworkers found more erosions with CT than with MRI. Whereas that study included joints with severe damage, the patients in the present study were selected on the basis of their having joints with MRI erosions that were radiographically occult, increasing the opportunity to demonstrate the specificity of radiographically invisible MRI erosions. The moderate sensitivities of MRI and, particularly, US obtained in the present study suggest the presence of more erosions than were detected by MRI (Figure 2) and US. However, because the sensitivity of MRI and US in this and several other studies [9-11,13,20-22] has been found to be much higher than that of radiography, we consider it acceptable that some minimal erosions are missed by MRI and US as long as the identified erosions are real. However, it should be emphasized that the sensitivities, specificities and accuracies reported in this study are study specific and not directly transferable to other patient cohorts. The moderate sensitivity of MRI found in this study suggests that the applied OMERACT RAMRIS definition of erosions [30] does not overestimate the number of erosions; we consider this to be of major importance, and so we believe that the present study further supports the future use of the OMERACT RAMRIS definition of bone erosion.

Overall, the sensitivity of US in detecting bone erosions in the present study was lower than the sensitivity of MRI. Several other studies have reported that the sensitivity of US is best at the most easily accessible joints (that is, the 2nd and 5th MCP joints), where visualization of the joint is possible from three aspects [20,22]. In this study we also achieved the highest sensitivity at these joints, but even in these joints there are certain bone surfaces that are not accessible to US assessment, contributing to the lower sensitivity of US. When looking at the joint surfaces accessible for US examination (that is, the dorsal and palmar aspects of all joints and the radial aspect of the 2nd and the ulnar aspect of the 5th MCP joint), US achieved markedly higher sensitivity (60%) compared with CT.

In two patients no erosions were detected on US, however, in all four healthy control individuals 'false positive' erosions were registered, whereas none of these were seen on CT, MRI and radiography. On US three control individuals had one erosion-like lesion each, whereas in the last control individual eight sites were registered with erosion-like lesions. It should be noted that the latter control individual developed a HLA-B27 positive arthritis one year later, which was treated with sulfasalazine. There was no tendency toward any specific regions in which erosion-like changes were seen on US. That erosion-like changes were observed in healthy control individuals in the present study is in agreement with previous US studies [20,21], even though the frequency was markedly higher in the present study. Furthermore, small well defined bone defects at the dorsal aspect of the metacarpal head have been reported in 37% of healthy control individuals [32]. Work on standardization of definitions of pathology in musculoskeletal US is being done in the setting of the European League Against Rheumatism (EULAR) Working Party for Ultrasound and OMERACT, and the definitions on erosions are not discordant with those used in the present study [33].

When finding pathological changes in healthy control individuals, using any diagnostic procedure, it should be considered whether the method is too sensitive, and our findings question whether the definition of US erosions used in the study is optimal. However, there is an inherent, usually divergent balance between the sensitivity and specificity of a test. In clinical trials, in which the diagnosis is established, a high sensitivity is often of fundamental importance. However, in a diagnostic setting high specificity may have the highest priority, because the diagnosis has important implications for classification and treatment.

Despite descriptions in the literature of CT findings in RA peripheral joints [12,26,34], CT is not a thoroughly validated method in RA, and use of CT as a standard comparator for better validated imaging methods such as MRI and US could therefore be questioned. However, high-resolution CT is the optimal radiographic method because it provides high-resolution tomographic direct visualization of calcified tissue, and CT is known from other skeletal conditions to be highly accurate. Thus, although CT findings may not represent the absolute truth, we found that comparison with CT provided very important validation of MRI and US findings.

Examination of the inter-reader agreement was not the objective of this study, and because previous papers have reported good inter-reader agreements for the readers of MRI (BE) [35] and US (MC-P) [31] involved in the present study, evaluations of magnetic resonance images and the US examination were done only once. CT, being less validated in RA, was evaluated by two readers (MØ and MH) in order to calculate the inter-observer agreement in reading CT images. The good inter-reader agreement of 90% is comparable with inter-observer agreements achieved in other studies with other imaging modalities [31,35].

The present study suggests that CT may be a very sensitive method for detecting early bone erosions, possibly even more so than MRI and US, but further studies (for example, on validity) are needed before any general recommendations on the use of CT in RA can be given.

## Conclusion

MRI and US exhibited high specificities in detecting bone erosions in RA MCP joints, even in radiographically non-eroded joints, when CT was used as the reference method. The moderate sensitivities of MRI and US indicate that even more erosions than were detected using MRI and US were present. Radiography had markedly lower sensitivity for bone erosions than CT, MRI and US.

The present study strongly indicates that bone erosions, detected by MRI and US in RA patients, represent loss of calcified tissue with cortical destruction, and therefore can be considered true bone erosions.

## Abbreviations

CT = computed tomography; MCP = metacarpophalangeal; MRI = magnetic resonance imaging; OMERACT = Outcome Measures in Rheumatology; RA = rheumatoid arthritis; RAMRIS = Rheumatoid Arthritis MRI Scoring System; US = ultrasonography.

## Authors' contributions

UMD participated in the study development and recruitment of patients, conducted data evaluation and statistical analysis, and prepared the manuscript draft. BE participated in the study development, performed the evaluation of magnetic resonance images, and was involved in patient recruitment. MC-P performed the ultrasonographic examinations. MH was involved in the CT scanning protocol and evaluated CT images. EN performed the evaluation of radiographs. MS participated in study development. JM was involved in the MRI scanning protocol and performed all MRI examinations. HT participated in study development and gave substantial input to the data evaluation and manuscript preparation. MØ participated in the study development, was involved in the CT and MRI scanning protocol, evaluated CT images, and gave substantial input to the data evaluation and manuscript preparation. All authors read and approved the final manuscript.

## Figures and Tables

**Table 1:** Characteristics of patients and control individuals

	Patients	Controls
Number	17	4
Sex (female/male)	13/4	3/1
Median age (years; range)	52 (33–78)	35.5 (34–57)
Median disease duration (years; range)	8 (4–22)	-
IgM rheumatoid factor (percentage seropositive)	82%	-

**Table 2:** Sensitivity, specificity and accuracy of radiography, MRI and US for bone erosions, with CT as reference

	MCP 2		MCP 3		MCP 4		MCP 5		Total
	Proximal	Distal	Proximal	Distal	Proximal	Distal	Proximal	Distal	
Quadrants with CT erosions	18	18	13	3	9	5	8	4	78
Quadrants with radiographic erosions	7	7	0	0	1	0	1	0	16
Quadrants with MRI erosions	19	11	16	0	7	2	8	1	64
Quadrants with US erosions	25	11	4	1	3	0	9	2	55
Radiography sensitivity	33%	39%	0%	0%	11%	0%	13%	0%	19%
Radiography specificity	96%	100%	100%	100%	100%	100%	100%	100%	100%
Radiography accuracy	69%	74%	69%	93%	81%	88%	83%	90%	81%
MRI sensitivity	83%	61%	85%	0%	78%	40%	75%	25%	68%
MRI specificity	83%	100%	83%	100%	100%	100%	94%	100%	96%
MRI accuracy	83%	83%	83%	93%	95%	93%	90%	93%	89%
US sensitivity	94%	33%	8%	0%	33%	0%	63%	25%	42%
US specificity	67%	79%	90%	97%	100%	100%	88%	97%	91%
US accuracy	79%	59%	64%	90%	86%	88%	83%	90%	80%

**In total, 336 MCP joint quadrants were evaluated. CT, computed tomography; MCP, metacarpophalangeal joint; MRI, magnetic resonance imaging; US, ultrasonography**.

**Table 3:** Sensitivity, specificity and accuracy of MRI and US for bone erosions in regions without radiographic erosions, with CT as the reference

	MCP 2		MCP 3		MCP 4		MCP 5		Total
	Proximal	Distal	Proximal	Distal	Proximal	Distal	Proximal	Distal	
Quadrants with CT erosions	12	11	13	3	8	5	7	4	63
Quadrants with MRI erosions	12	6	16	0	7	2	7	1	51
Quadrants with US erosions	18	5	4	1	2	0	8	2	40
MRI sensitivity	75%	55%	85%	0%	88%	40%	71%	25%	65%
MRI specificity	87%	100%	83%	100%	100%	100%	94%	100%	96%
MRI accuracy	83%	86%	83%	93%	98%	93%	90%	93%	90%
US sensitivity	92%	0%	8%	0%	25%	0%	57%	25%	30%
US specificity	70%	79%	90%	97%	100%	100%	88%	97%	92%
US accuracy	77%	54%	64%	90%	85%	88%	83%	90%	80%

320 MCP joint quadrants were evaluated. CT, computed tomography; MCP, metacarpophalangeal joint; MRI, magnetic resonance imaging; US, ultrasonography.
